# Antimicrobial Resistance of *Listeria monocytogenes* Strains Isolated from Humans, Animals, and Food Products in Russia in 1950–1980, 2000–2005, and 2018–2021

**DOI:** 10.3390/antibiotics10101206

**Published:** 2021-10-04

**Authors:** Pavel A. Andriyanov, Pavel A. Zhurilov, Elena A. Liskova, Tatyana I. Karpova, Elena V. Sokolova, Yulia K. Yushina, Elena V. Zaiko, Dagmara S. Bataeva, Olga L. Voronina, Ekaterina K. Psareva, Igor S. Tartakovsky, Denis V. Kolbasov, Svetlana A. Ermolaeva

**Affiliations:** 1 Federal Research Center for Virology and Microbiology, Branch in Nizhny Novgorod, 603950 Nizhny Novgorod, Russia; andriyanovpvl@gmail.com (P.A.A.); Zhurilov95@bk.ru (P.A.Z.); liskovaea@mail.ru (E.A.L.); sokol.e1ena@yandex.ru (E.V.S.); ekaterinapsareva@gmail.com (E.K.P.); 2 Gamaleya National Research Centre for Epidemiology and Microbiology, 123098 Moscow, Russia; dragovtceva@yandex.ru (T.I.K.); olv550@gmail.com (O.L.V.); itartak@list.ru (I.S.T.); 3 Federal Scientific Centre for Food Systems n.a. V.M. Gorbatov, 109316 Moscow, Russia; yshinauk@mail.ru (Y.K.Y.); zaiko@fncps.ru (E.V.Z.); d.bataeva@fncps.ru (D.S.B.); 4 Federal Research Center for Virology and Microbiology, 601125 Volginsky, Russia; kolbasovdenis@gmail.com

**Keywords:** antibiotic resistance, food pathogen, *Listeria monocytogenes*

## Abstract

Susceptibility of 117 *L. monocytogenes* strains isolated during three time periods (1950–1980; 2000–2005, and 2018–2021) to 23 antibiotics was tested by the disk diffusion method. All strains were sensitive to aminoglycosides (gentamicin, kanamycin, neomycin, streptomycin), glycopeptides (vancomycin and teicoplanin), clarithromycin, levofloxacin, amoxicillin/clavulanic acid, and trimethoprim/sulfamethoxazole. Resistance to clindamycin was observed in 35.5% of strains. Resistance to carbapenems, imipenem and meropenem was found in 4% and 5% of strains, respectively. Resistance to erythromycin, penicillin G, trimethoprim, and ciprofloxacin was found in 4%, 3%, 3%, and 2.5% of strains, respectively. Resistance to tylosin, ampicillin, enrofloxacin, linezolid, chloramphenicol, and tetracycline was found in less than 2%. Three strains with multiple antibiotic resistance and 12 strains with resistance to two antibiotics were revealed. Comparison of strains isolated in different time periods showed that the percentage of resistant strains was the lowest among strains isolated before 1980, and no strains with multiple antibiotic resistance were found among them. Statistical analysis demonstrated that the temporal evolution of resistance in *L. monocytogenes* has an antibiotic-specific character. While resistance to some antibiotics such as ampicillin and penicillin G has gradually decreased in the population, resistance to other antibiotics acquired by particular strains in recent years has not been accompanied by changes in resistance of other strains.

## 1. Introduction

The foodborne pathogen *Listeria monocytogenes* causes listeriosis, a serious sometimes fatal disease with such manifestations as meningitis, meningoencephalitis, rhombencephalitis, and abortion [1]. Elderly and immuno-compromised individuals, pregnant women, and newborns are groups at risk for listeriosis. While incidence of listeriosis is relatively low, the fatality rates are quite high reaching 20–25% of patients [2,3,4]. The incidence of pregnancy-related listeriosis ranges from 4 to 25 per 100,000 births, and the mortality rates reach 30% or more. [5,6,7].

The final outcome of listeriosis depends on the early administration of antibiotics [6,8]. The importance of selecting the correct antibiotic with bactericidal action requires monitoring the spread of antibiotic resistance among *L. monocytogenes* strains occurring in a particular region. Being a foodborne pathogen of zoonotic origin, *L. monocytogenes* is included in the list of pathogens requiring compulsory annual monitoring for spreading antibiotic resistance among strains of a human, animal and food origin [9,10].

The species *L. monocytogenes* is classified into four phylogenetic lineages [11]. The lineages I and II contribute to the majority of human and animal cases of listeriosis, and lineage II strains prevailed among food isolates, while the lineage III and IV are relatively rare among all sources [11,12]. The lineage I serotype 4b strains are the most frequent causative agents of outbreaks among humans and severe neurological disease in domestic animals [13,14]. Lineage II strains and particularly the clonal complex CC7 are historically prevalent in the territory of Russia including pristine environments with natural foci of the infection [15,16,17,18].

*L. monocytogenes* is naturally resistant to fosfomycin, fusidic acid and to majority of second- and third-generation cephalosporins (cefetamet, cefotaxime, ceftriaxone, cefuroxime etc.) [19,20]. Besides these exceptions, *L. monocytogenes* is susceptible to clinically-relevant classes of antibiotics active against Gram-positive bacteria. Ampicillin/amoxicillin alone or in combination with gentamicin remains the treatment of choice [19]. Second-line agents for listeriosis treatment include trimethoprim/sulfamethoxazole, erythromycin, vancomycin, and the fluoroquinolones [21,22].

Acquired antibiotic resistance of *L. monocytogenes* strains varied widely depending on the source and year of isolation, and the geographic origin. A noticeable increase of acquired antibiotic resistance was observed in recent years [23,24]. The emergence and spread of resistant strains represent a serious threat for human health and require monitoring of changes in *L. monocytogenes* antibiotic resistance.

The aim of this work was to establish antibiotic resistance profiles of *L. monocytogenes* strains isolated from humans, animals, and food products in the European part of Russia.

## 2. Results

### 2.1. Strain Characterization

**Figure 1:**
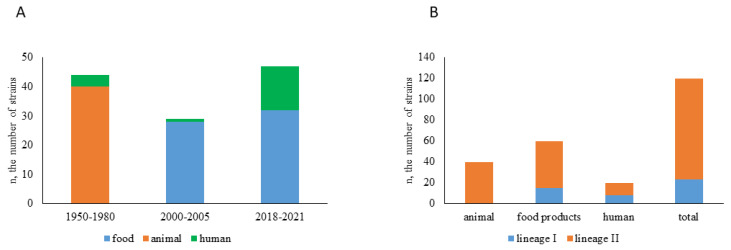
Strains studied. (**A**)—strain distribution relative to the year of isolation. (**B**)—strain distribution relative to the phylogenetic lineage.

A total of 117 strains were included into the study. All strains were isolated in the European part of Russia in three time periods including 1950–1980 (*n* = 45), 2000–2005 (*n* = 27), and 2018–2021 (*n* = 45) (Figure 1 and Table S1). Strains of human clinic, animal, and food origin (*n* = 20, *n* = 39 and *n* = 58, respectively) were studied. The distribution of strains relatively to the source was unequal with all animal strains isolated before 2000 (1950–1980), and all food strains isolated after 2000 (2001–2021). The uneven distribution was mainly due to the historical change in the role of *L. monocytogenes* as an infectious agent and the corresponding shift in the focus of attention to *L. monocytogenes* isolation. Before 1980, listeriosis was mainly considered as an animal disease although human clinical cases were registered [25,26]. The regulation law was accepted in Russia in 2002 to monitor *L. monocytogenes* in food products [27]. The importance of *L. monocytogenes* as an animal pathogen decreased significantly in Russia after 1980 which might be partly due to wide introduction of antibiotics and partly due to changes in farming activities.

The studied strains were characterized with multilocus sequence typing ([12], see Table S1). All strains belonged to the phylogenetic lineages I and II, there were no strains that belonged to the lineages III or IV (Figure 1B). The lineage II strains prevailed (97 vs. 23 strains for lineage II and lineage I, respectively) which is in line with the previously demonstrated prevalence of lineage II strains in the territory of the European part of Russia [15,16,17,18].

### 2.2. Frequency of Antibiotic Resistance among Strains Tested

**Figure 2:**
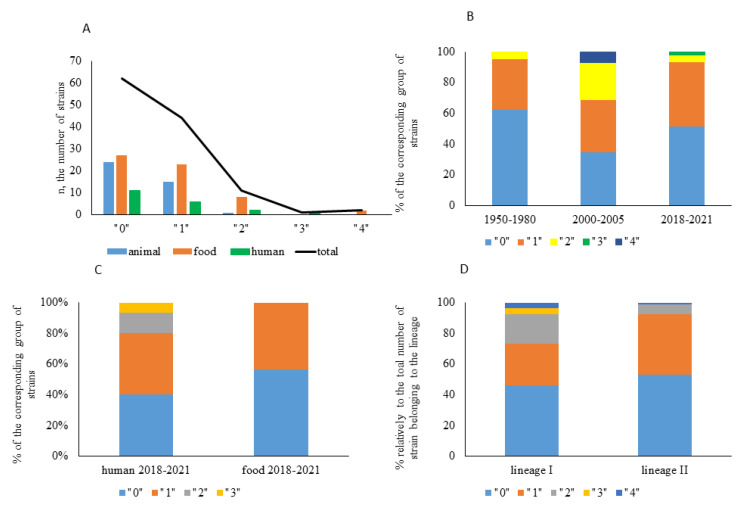
Antibiotic resistant strain distribution. Resistance to 23 antibiotics was tested “0”–strains sensitive to all antibiotics tested; “1”–strains resistant to one antibiotic; “2”–strains resistant to two antibiotics; “3”–strains resistant to three antibiotics; “4”–strains resistant to four antibiotics. (**A**)—strain distribution relative to the source. (**B**)—strain distribution relative to the year of isolation; (**C**)—distribution of strains isolated in 2018–2021 from humans and food products; (**D**)—percentage of strains resistant to 0, 1, 2, 3, or 4 antibiotics among strains of the I and II phylogenetic lineages.

In total, 59 strains (51% of the total amount of strains) were resistant to one or more antibiotics (Figure 2). Among them, 44 strains were resistant to one antibiotic. Twelve, one, and two strains demonstrated resistance to two, three, and four antibiotics, respectively.

Among strains of animal origin, 15 strains (37.5% of animal isolates) were resistant to one antibiotic. Two strains (5% of animal isolates) were resistant to two antibiotics, and there were no strains resistant to three or four antibiotics. Among strains of food origin, 27 strains (45% of food isolates) were sensitive to all antibiotics, 23 strains (38% of food isolates) were resistant to one antibiotic, and 10 strains (17%) were resistant to more than one antibiotic. The only two strains resistant to four antibiotics were of the food origin. Among strains of human clinical origin, 11 strains (55% of human isolates) were sensitive to all antibiotics tested, six strains (30%) were resistant to one antibiotic, and three strains (15% of strains of human origin) were resistant to two or three antibiotics (Figure 2A).

The analysis of resistance among strains isolated in different time periods (1950–1980, 2000–2005 and 2018–2021; Figure 2B) showed that the percentage of resistant strains was the lowest among strains isolated before 1980. Percentage of strains with resistance to more than one antibiotic was highest among isolates obtained in 2000–2005. All human isolates obtained before 1980 (*n* = 4) were sensitive to all antibiotics tested. Among human isolates obtained in 2018–2021, six strains (40% of human isolates obtained in 2018–2021) were sensitive to all antibiotics, while 40%, 13%, and 7% were resistant to one, two, or three antibiotics, respectively. Among food isolates obtained in the same period, 56% were sensitive to all antibiotics, 44% were resistant to 1 antibiotic, and there were no strains with multiple resistance (Figure 2C). All strains with multiple antibiotic resistance were isolated after 2000.

When distribution of resistant strains was analyzed according to their phylogenetic positions, a higher percentage of strains with multiple resistance was observed among lineage I strains comparatively to lineage II strains (Figure 2D).

The antibacterial resistance index (ARI) of strains isolated in different periods was calculated (Table 1). ARI scores above 0.2 suggest that the selective pressure due to environment contamination with antibiotics can promote dissemination of resistance determinants [28,29]. ARI scores for all studied groups were below 0.1 suggesting relatively low environment contamination. The ARI scores for strains isolated in 1950–1980 were 3.3 and 1.5 times lower than for strains isolated in 2002–2005 and 2018–2021, respectively. Taken together, the results demonstrated that the percentage of antibiotic resistant strains increased after 2000.

### 2.3. Characterization of Antibiotic Resistance

In total, 23 antibiotics belonging to aminoglycosides, β-lactams, macrolides, quinolones, and some other classes were tested (Table 1). All strains were sensitive to aminoglycosides (gentamicin, kanamycin, neomycin, streptomycin) and glycopeptides (vancomycin and teicoplanin). Other antibiotics effective against all strains tested were clarithromycin, levofloxacin, and combinations of amoxicillin and clavulanic acid, and trimethoprim and sulfamethoxazole. Resistance to clindamycin was observed in the largest number of strains (*n* = 45; 35.5% of the total number of strains). Resistance to carbapenems imipenem and meropenem was found in five (4%) and six (5%) strains, respectively. Resistance to erythromycin was found in five strains (4%). four (3%) and four (3%) strains were resistant to penicillin G and trimethoprim. Three strains (2.5%) were resistant to ciprofloxacin. Two strains (1.7%) were resistant to tylosin. Single strains (less than 1% of the total number strains) were resistant to ampicillin, enrofloxacin, linezolid, chloramphenicol, and tetracycline.

Among 44 strains resistant to one antibiotic only, resistance to clindamycin was found in 33 strains, and 11 strains were resistant to one antibiotic that was not clindamycin (Table 2). Twelve strains were resistant to two antibiotics, and three strains with multiple antibiotic resistance (MAR, resistance to ≥3 antibiotics) were found including one strain resistant to three antibiotics, and two strains resistant to four antibiotics (Table 3). Resistance to clindamycin was found in 10 strains resistant to two antibiotics and all three MAR strains. Resistance to β-lactams, and particularly, resistance to carbapenems was found in five of 15 strains including two MAR strains. Four strains including two MAR strains demonstrated resistance to trimethoprim. All but two strains with resistance to two or more antibiotics were isolated after 2000. The strain isolated from pigs in 1967 was resistant to penicillin G and enrofloxacin demonstrating the preexisting natural resistance to the quinolone antibiotic as enrofloxacin was under clinical studies in the 1980s and approved in the 1990s [30,31].

Two MAR strains belonged to the phylogenetic lineage II (CC8 and CC9), and the third MAR strain belonged to the phylogenetic lineage I (CC6). Among strains with resistance to two antibiotics, similar resistance patterns (resistance to clindamycin and imipenem, and resistance to clindamycin and trimethoprim) were observed twice in phylogenetically distant strains belonging to distinct clonal complexes and phylogenetic lineages. Taken together, the results obtained demonstrated an independent development of multiple resistance in strains of different origin.

### 2.4. Temporal Changes in Antibiotic Resistance Patterns

**Figure 3:**
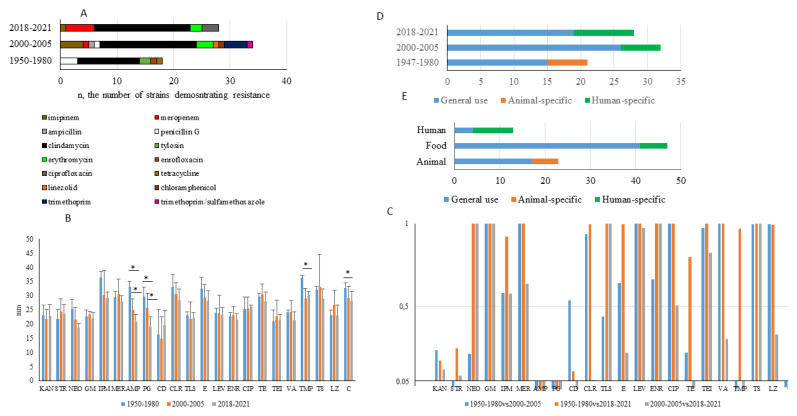
Temporal changes in antibiotic resistance patterns. (**A**)—resistance distribution relative to the year of isolation; (**B**)—inhibition zones; mean values in mm are shown and compared using a *t*-test; *—*p* < 0.05; (**C**)—χ^2^ testing of hypothesis on similarity of data arrays describing growth inhibition zone for strains isolated in different time periods, the cut point is 0.05 (the abscissa axis intersects the ordinate axis at the point 0.05); (**D**)—distribution of antibiotic resistance depending on antibiotic applicability (general use, human only, or animal only) relative to the year of isolation; (**E**)—distribution of antibiotic resistance depending on antibiotic applicability relative to the source of strain isolation. KAN—kanamycin, STR—streptomycin, NEO—neomycin, GM—gentamicin, IPM—imipenem, MER—meropenem, AMP—ampicillin, PG—penicillin G, CD—clindamycin, CLR—clarithromycin, TLS—tylosin, E—erythromycin, LEV—levofloxacin, ENR—enrofloxacin, CIP—ciprofloxacin, TE—tetracycline, TEI—teicoplanin, VA—vancomycin, TMP—trimethoprim, TS—trimethoprim/sulfamethoxazole, LZ—linezolid, C—chloramphenicol.

A shift was observed in resistance patterns between strains isolated in different periods (Figure 3A). All strains resistant to penicillin G, ampicillin, tetracycline, tylosin, and chloramphenicol were isolated in 1950–1980 or 2000–2005 but not later. Resistance to carbapenems and ciprofloxacin was found in strains isolated after 2000 only. Clindamycin-resistant strains were found among isolates obtained in all periods studied.

To obtain more evidence on the temporal evolution of antibiotic resistance, we calculated mean values of the inhibition zones and performed statistical analysis of pairwise differences in the susceptibility of randomly selected strains isolated in the time periods of 1950–1980, 2000–2005 and 2018–2021. The mean values of the inhibition zones and statistical testing of data arrays showed a noticeable difference in susceptibility to penicillin G and ampicillin between all pairwise compared periods (Figure 3B,C). The decrease in resistance to trimethoprim/sulfamethoxazole was found in strains isolated in 2018–2021 comparative to earlier periods. Despite the appearance of strains with resistance to carbapenems after 2000, the mean resistance of the majority of the strains was maintained at a stable level. Comparison of stably effective antibiotics such as aminoglycosides or glycopeptides did not reveal temporal differences.

Most antibiotics studied are applied in both human and veterinary medicine. Nonetheless, some antibiotics are used exclusively for humans (imipenem, meropenem, ciprofloxacin, linezolid) or animals (tylosin, enrofloxacin). Resistance to animal-specific antibiotics was observed mainly among strains of animal origin (Figure 3E). Resistance to antibiotics of general use including ampicillin and penicillin G was observed consistently among strains from different sources and at different time periods.

## 3. Discussion

In this work, we tested the susceptibility of 117 *L. monocytogenes* strains isolated in Russia to 23 antibiotics widely used in medical and veterinary practice. For all but one antibiotic tested, the occurrence of antibiotic resistance was low and did not exceed 5% of strains tested. The only exception was clindamycin; 45 of 117 strains were resistant to clindamycin, and at least 33 strains demonstrated intermediate resistance (data not shown). Here our data are in line with results obtained by other authors. In particular, 26.6% *L. monocytogenes* isolated from sliced cheese and ham in Brazil, 81% of *L. monocytogenes* isolated from raw milk, milking equipment and dairy workers in Egypt, and 90% *L. monocytogenes* isolated from ready-to-eat products of animal origin in Spain were resistant to clindamycin [32,33,34].

After the 1960s, ampicillin or amoxicillin in combination with aminoglycosides were considered as the treatment of choice for listeriosis [21,22,35]. Later publications supported this view. No ampicillin resistant strains and only one gentamicin-resistant strain were found among 544 strains isolated in Germany [36]. Less than 3% of the strains showed resistance to ampicillin and gentamicin among 118 *L. monocytogenes* isolates from meat in Spain [37]. Our results demonstrate low occurrence of ampicillin/amoxicillin and aminoglycoside resistant strains in Russia. Still, other works reported a high percentage of ampicillin-resistant strain suggesting amoxicillin/clavulanic acid as a better choice [36,38].

Among second-line agents for listeriosis treatment tested in this work, vancomycin was active against all strains studied. Another glycopeptide, teicoplanin, was also fully active. No strains resistant to trimethoprim/ sulfamethoxazole in contrast to four strains resistant to trimethoprim alone support the view of co-trimoxazol as an effective antibiotic for listeriosis treatment. Levofloxacin was active against all strains but as for other fluoroquinolones besides levofloxacin, 2.5% and 3% of all strains demonstrated resistance to ciprofloxacin and enrofloxacin, respectively. Our data are in line with other studies revealing from 0 to 13% of ciprofloxacin-resistant and less than 3% of enrofloxacin-resistant strains among *L. monocytogenes* isolated in some European countries [34,35,37]. Nonetheless, in other countries the situation with resistance to fluoroquinolones might be different. Therefore, 38.37% and 56.97% *L. monocytogenes* isolated in Iran were resistant to ciprofloxacin and enrofloxacin, respectively, [38]. Thirteen of 50 strains isolated in Spain were resistant to ciprofloxacin [34]. Erythromycin is another antibiotic of choice in cases of allergy to β-lactams; 4% of studied strains were resistant to erythromycin which is relatively higher than in works analyzing *L. monocytogenes* strains isolated in other European countries in recent years [36,37,39].

Five strains demonstrated resistance to imipinem and/or meropenem but were sensitive to ampicillin that was unexpected. Resistance to carbapenems among Gram-positive bacteria usually develops through mutations in penicillin-binding proteins (PBPs) [40]. We suppose that the resistance in our strains may be associated with PBP mutations that gives rise to carbapenems resistance but doesn’t affect the ampicillin (penams) susceptibility. To confirm this hypothesis, further investigations are needed.

Only three strains with multiple antibiotic resistance, and only 12 strains with resistance to two antibiotics were revealed among strains of the collection. We suggest that the low occurrence of MAR strains is due to the high proportion of strains isolated in 1950–1980. The temporal evolution of antibiotic resistance and absence of acquired resistance in *L. monocytogenes* before 1989 was demonstrated by Morvan et al. when strains isolated from humans from 1926 up to 2007 were compared [23]. The temporal evolution of antibiotic resistance was observed in German strains isolated from milk products in 1994 and 2009/2010 [24]. Our data demonstrated that the temporal evolution of antibiotic resistance has an antibiotic-specific character. While resistance to some antibiotics such as ampicillin and penicillin G gradually decreases in the population as a whole, the appearance of resistance to other antibiotics by particular strains is not accompanied by changes in population resistance.

The first *L. monocytogenes* MAR strains were described in European countries after [23,41,42]. MAR strains described in this work were isolated in 2005–2019 suggesting that the spread of multidrug resistance in Russia has similar or even slower dynamics as in other countries (Table 3). The noticeable increase in the spread of antibiotic determinants was observed by some authors with the incidence of MAR strains reaching 30% or more in the last 10 years especially among environmental, animal and food isolates [43,44,45]. Our results on MAR strains’ occurrence in food products and clinical cases in 2018–2021 do not support this observation for *L. monocytogenes* isolated in Russia. Nonetheless, the trend of increasing acquired resistance in *L. monocytogenes* that now concerns a number of countries might affect the whole world in future.

Taken together, the results obtained demonstrated a relatively low incidence of antibiotic resistance among *L. monocytogenes* strains in Russia with exceptionally low or total absence of strains resistant to first line antibiotics used to treat listeriosis. Nonetheless, our data demonstrated an increase of antibiotic resistance since 2000 and, in particular, the appearance of MAR strains among human isolates that indicate the need for regular monitoring of antibiotic resistance among newly isolated *L. monocytogenes* strains.

## 4. Materials and Methods

### 4.1. Bacterial Strains and Characterization Methods

In total, 117 *L. monocytogenes* strains were included in the study. The strains have been collected since the 1950s. Each strain was isolated from an individual source comprising human and veterinary patients, meat, fish, dairy and other food products, and food processing environment (Table S1). The majority of strains were isolated in the European part of Russia, mainly at the territory of Moscow, Tula, and Tver regions (see Table S1). All strains were identified as *L. monocytogenes* by bacteriological, biochemical, and serological methods. Briefly, typical *Listeria* spp. colonies were characterized on the ability to hydrolyze sugars, including rhamnose, xylose, and methyl-D-mannopyranoside, haemolyitic activity using the agar plates supplemented with the sheep blood, induction of the phospholipase activity [27]. Serological characterization was performed for strains isolated before 2000 using specific antisera. Strains were kept lyophilized (strains isolated in 1950–1980) or frozen at −86°C (strains isolated in 2000 and latter) in collections of the Federal Research Center for Virology and Microbiology, Gamaleya National Research Centre of Epidemiology and Microbiology, and Gorbatov Federal Centre of Food Systems. When the work started, strains were revitalized and characterized bacteriologically and biochemically to support their identities. Characterization by the multilocus-locus sequence typing method using the scheme suggested by Ragon et al. [12] in modification of Voronina et al. [17] was performed and the data are represented in the Table S1 and at https://bigsdb.pasteur.fr/listeria/listeria.html. Characterized strains were kept frozen, until antibiotic resistance testing started.

### 4.2. Antibiotic Susceptibility Testing

*L. monocytogenes* strains kept frozen were thawed, plated on Brain Heart Infusion agar (BHI, BD, Disco) and incubated at 37 °C for 24 h. Antibiotic susceptibility testing was conducted using the disc diffusion method as recommended by the European Committee on Antimicrobial Susceptibility Testing (EUCAST). Briefly, 5–6 overnight colonies were suspended in 1 mL of 0.9% NaCl solution and turbidity was adjusted to a 0.5 McFarland. The suspension was used to inoculate Mueller–Hinton agar (HiMedia, India). Disks with antibiotics (NICF, LLC; St. Petersburg, Russia; HiMedia India) were transferred on the agar using a disc dispenser. Plates were incubated at 37 °C for 24 h. The inhibition zones were measured to the nearest mm. Data interpretation was performed according to the EUCAST criteria [46]. Missing breakpoints if the EUCAST guidelines give no resistance criteria for *Listeria* were complemented by those recommended for *Staphylococcus aureus*, *Enterococcus* spp. by CLSI standards [47].

In total, 23 antibiotics used for listeriosis treatment or the alleviation of other Gram-positive bacteria in human and veterinary medicine were tested including penicillin G (PG-10 μg), ampicillin (AP-10 μg), amoxicillin/clavulanic acid (AKK-20/10 μg), imipenem (IMP-10 μg), meropenem (MRP-10 μg), kanamycin (KAN-30 μg), gentamicin (GM-10 μg), streptomycin (STR-10 μg), neomycin (NEO-30 μg), ciprofloxacin (CIP-5 μg), levofloxacin (LEV-5 μg), enrofloxacin (ENR-10 μg), erythromycin (E-15 μg), tylosin (TLS-15 μg), clarithromycin (CLR-15 μg), clindamycin (CD-2 μg), vancomycin (VA-5 μg), teicoplanin (TEI-30 μg), tetracycline (TE-30 μg), trimethoprim (TMP-5 μg), trimethoprim/sulfamethoxazole (TS-1.25 μg/23.75 μg), linezolid (LZ-10 μg), and chloramphenicol (C-30 μg).

### 4.3. Antibiotic Resistance Index (ARI)

The antibiotic resistance index (ARI) is used to index the frequency with which antibiotic resistance occurs among isolates obtained from a particular sample [46]. The following formula was used to calculate ARI:

ARI = A/NY

where A is a total number of resistance determinants recorded in the population, N is the number of isolates in the population, and Y is the total number of antibiotics tested [28,29].

### 4.4. Statistics

Three independent experiments were performed for each strain and the mean values were used to prescribe strain resistance. Mean values were compared using a *t*-test. A *p*-value below 0.05 was considered significant. To test temporal and source-specific differences in resistance patterns, *L. monocytogenes* strains from different groups were randomly selected with an equal distribution and choice without returning and data arrays describing growth inhibition zones were compared using the chi-square test.

## Figures and Tables

**Table 1:** The antibacterial resistance index (ARI).

Period of Isolation	Source of the Strain			Phylogenetic Position		All Collection
	Human	Food	Animal	Lineage I	Lineage II	
1950–1980	0.016	n.a.	0.019	0	0	0.017
2000–2005	0.055	0.051	n.a.	0.043	0.051	0.039
2018–2021	0.024	0.019	n.a.	0.052	0.020	0.027
total	0.031	0.039	0.019	0.043	0.056	0.031

n.a.–not applicable.

**Table 2:** Antibiotics tested.

Class	Antibiotic	Total Number of Resistant Strains	Number of Strains with Resistance to Only One Antibiotic
Aminoglycosides			
	Gentamicin	0	0
	Kanamycin	0	0
	Neomycin	0	0
	Streptomycin	0	0
			
β-Lactams:Penicillins			
	Penicillin G	4	2
	Ampicillin	1	0
	Amoxicillin/clavulanic acid	0	0
β-Lactams:Carbapenems			
	Imipenem	5	1
	Meropenem	6	2
Macrolides			
	Clarithromycin	0	0
	Tylosin	2	2
	Erythromycin	5	3
Lincosamides			
	Clindamycin	45	33
Quinolones			
	Ciprofloxacin	3	1
	Levofloxacin	0	0
	Enrofloxacin	1	0
Glycopeptides			
	Teicoplanin	0	0
	Vancomycin	0	0
Other antibiotics			
	Trimethoprim	4	0
	Trimethoprim/sulfamethoxazole	1	0
	Linezolid	1	0
	Chloramphenicol	1	0
	Tetracycline	1	0

**Table 3:** *L. monocytogenes* strains resistant to two and more antibiotics.

N	Strain	Resistance	CC/Lineage	Year	Source
1	178-P	PG -ENR	CC7/II	1967	pig
2	3880	CD-TE	CC7/II	1970	pig
3	24-T	CD-IPM	CC37/II	2005	dairy product
4	14-2	CD-IPM	CC6/I	2001	fish
5	35-T	CD-TR	CC37/II	2005	dairy product
6	134/3	CD-TR	CC2/I	2005	dairy product
7	1300	CD-PG	CC1/I	2005	dairy product
8	44	CD-AMP	CC7/I	2002	meat
9	98/20	CD-LZ	CC9/II	2005	dairy product
10	13215	CD-E	CC59/I	2005	dairy product
11	UH18	CD-CIP	CC155/II	2019	perinatal, blood
12	H67-1	IMP-MER	CC6/I	2019	clinical, adult
13	UH19	CD–MER-CIP	CC6/I	2019	perinatal, blood
14	129/3	CD–MER–IPM-TMP	CC9/II	2005	dairy products
15	114/31	CD–E–C-TMP	CC8/II	2005	dairy products

PG = Penicillin G; ENR = Enrofloxacin; CD = Clindamycin; TE = Tetracycline; IPM = Imipenem; TMP = Trimethoprim; AMP = ampicillin; LZ = Linezolid; E = Erythromycin; CIP = Ciprofloxacin; MER = Meropenem; and C = Chloramphenicol.

## Data Availability

Data is contained within the article and supplementary material.

## Supplementary Materials

The following are available online at https://www.mdpi.com/article/10.3390/antibiotics10101206/s1, Table S1: Bacterial strains used in the study.
