# Supplementary material for: Antimicrobial Resistance of Listeria monocytogenes Strains Isolated from Humans, Animals, and Food Products in Russia in 1950–1980, 2000–2005, and 2018–2021
Source: Antibiotics (Basel). 2021 Oct 4;10(10):1206. doi: 10.3390/antibiotics10101206 (PMC8532776; doi:10.3390/antibiotics10101206)
Supplement: Supplementary file 1 [file antibiotics-10-01206-s001.zip › antibiotics-1377683-supplementary.pdf]

antibiotics

MDPI

Article

Antimicrobial Resistance of *Listeria monocytogenes* Strains Isolated from Humans, Animals and Food Products in Russia in 1950–1980, 2000–2005, and 2018–2021

Pavel A. Andriyanov [1], Pavel A. Zhurilov [1], Elena A. Liskova [1], Tatyana I. Karpova [2], Elena V. Sokolova [1], Yulia K. Yushina [3], Elena V. Zaiko [3], Dagmara S. Bataeva [3], Olga L. Voronina [2], Ekaterina K. Psareva [1], Igor S. Tartakovsky [2], Denis V. Kolbasov [4] and Svetlana A. Ermolaeva [1,2,*]

1 Federal Research Center for Virology and Microbiology, Branch in Nizhny Novgorod, 603950 Nizhny Novgorod, Russia; andriyanovpvl@gmail.com (P.A.A.); Zhurilov95@bk.ru (P.A.Z.); liskovaea@mail.ru (E.A.L); sokol.e1ena@yandex.ru (E.V.S.); ekaterinapsareva@gmail.com (E.K.P.)
2 Gamaleya National Research Centre for Epidemiology and Microbiology, 123098 Moscow, Russia; dragovtceva@yandex.ru (T.I.K.); olv550@gmail.com (O.L.V.); itartak@list.ru (I.S.T.)
3 Federal Scientific Centre for Food Systems n.a. V.M. Gorbatov, 109316 Moscow, Russia; yshinauk@mail.ru (Y.K.Y); .zaiko@fncps.ru (E.V.Z.); d.bataeva@fncps.ru (D.S.B.)
4 Federal Research Center for Virology and Microbiology, 601125 Volginsky, Russia; kolbasovdenis@gmail.com
* Correspondence: drermolaeva@mail.ru; Tel.: +7-909-939-9612

Supplementary Materials

Table S1. Bacterial strains used in the study.

						KAN	S	Neo	CN	AKK	IPM	MRP	AMP	P	CD	CLR	TLS	E	LE	ENR	CIP	TE	TEI	VA	TR	SXT	RIF	LZ	C
**BIGSdb ID**	**Strain**	**Lineage**	**Year**	**Region**	**CC**	**General-Use**	**General-Use**	**Human**	**General-Use**	**General-Use**	**Human**	**Human**	**General-Use**	**General-Use**	**General-Use**	**General-Use**	**Animal**	**General-Use**	**General-Use**	**Animal**	**Human**	**General-Use**	**Animal**	**Human**	**General-Use**	**General-Use**	**Human**	**General-Use**	
**FOOD**																													
1	74-T	II	2005	Tula	CC 18	24	27	17	22	30	31	30	21	21	12	32	20	31	23	24	24	32	22	23	29	36	29	24	23
2	31-T	II	2005	Tula	CC 315	27	29	21	22	28	33	32	26	23	14	32	23	31	26	24	28	32	23	26	28	48	23	25	24
3	8712	II	2005	Tula	CC 8	28	27	24	23	17	34	29	24	21	23	30	20	29	30	30	31	31	20	23	19	32	25	27	25
4	98/20	II	2005	Tula	CC 9	21	27	19	24	27	31	31	25	25	11	26	20	29	20	22	21	25	21	20	27	44	18	20	27
5	L.mo 1300	II	2005	Tula	CC 1	22	28	22	21	32	28	30	26	12	12	26	14	26	23	22	27	24	20	24	28	42	28	22	20
6	129/3	II	2005	Tula	CC 9	22	24	20	24	28	20	25	20	20	9	25	20	25	20	20	22	22	22	22	0	36	26	18	22
7	114/31	II	2005	Tula	CC 8	14	22	22	23	16	34	27	25	21	12	17	20	15	25	25	25	25	16	16	0	28	27	9	18
8	13215	II	2005	Tula	CC 59	22	20	20	23	21	30	30	22	22	10	27	21	20	20	20	24	25	18	21	28	34	27	14	9
9	28	II	2000	-	CC 9	22	25	18	24	22	30	30	22	22	12	27	17	26	22	18	22	24	22	22	25	28	24	20	14
10	56-T	II	2005	Tula	CC 315	22	25	16	24	32	30	30	25	22	12	33	23	31	27	26	30	28	22	25	32	36	32	24	20
11	114/26	II	2005	Tula	CC 37	19	21	17	22	30	28	27	21	23	14	27	21	26	21	22	23	34	20	22	22	48	29	22	24
12	24-T	II	2005	Tula	CC 37	18	21	19	23	35	24	26	22	19	12	28	18	24	20	20	22	31	20	23	22	32	28	22	22
13	35-T	II	2005	Tula	CC 37	17	21	18	25	25	29	27	21	22	14	27	17	26	21	18	22	32	18	20	23	44	23	20	22
14	134/3	I	2005	Tula	CC 2	15	20	16	22	20	26	24	21	18	11	24	15	23	21	21	24	30	20	22	18	42	23	20	20
15	14	II	2002	Moscow,	CC 8	26	24	28	19	44	36	34	28	35	17	38	26	38	28	26	34	33	28	26	44	36	36	30	20
16	33	II	2002	Moscow,	CC 7	18	20	17	21	30	25	30	25	25	16	30	21	32	20	22	22	28	22	22	29	28	30	22	30

17	37	I	2002	Moscow,	CC 1	20	18	20	23	30	30	30	24	25	17	25	20	30	22	17	20	28	22	24	36	34	28	24	22
18	42	II	2002	Moscow,	CC 20	20	17	20	21	28	30	30	32	31	17	34	25	32	25	20	20	28	22	24	30	36	34	24	24
19	44	II	2002	Moscow,	CC 7	24	15	28	22	42	40	40	14	43	12	46	30	44	30	23	32	34	26	32	48	48	40	38	24
20	47	I	2002	Moscow,	CC 1	26	30	30	22	32	30	28	34	40	14	33	30	42	22	28	25	46	32	24	42	32	27	20	38
21	14=1	I	2001	Sankt-Petersburg	CC 6	22	28	24	23	38	42	35	34	36	24	40	28	38	27	26	32	45	30	30	40	44	36	33	20
22	14=2	I	2001	Sankt-Petersburg	CC 6	25	30	30	24	36	17	34	33	32	14	34	18	30	24	35	28	34	26	30	44	40	34	26	33
23	9	II	2002	Moscow,	CC 7	23	25	21	21	30	30	29	23	23	12	28	20	29	22	20	23	26	20	24	27	33	27	22	26
24	K-3	II	2003	Moscow,	CC 5	21	23	21	24	26	32	27	21	17	20	27	21	28	21	18	24	24	20	23	28	42	27	22	22
25	19	I	2002	Moscow,	CC 1	21	26	19	23	30	37	28	22	25	15	34	24	32	25	23	28	35	22	25	37	36	32	27	22
26	22	II	2002	Moscow,	CC 121	25	34	25	23	44	35	40	34	38	23	40	30	40	30	28	30	34	32	30	32	42	40	30	27
27	29	II	2002	Moscow,	CC 121	20	23	21	24	27	27	28	23	18	18	27	18	27	23	22	24	22	20	22	27	37	27	22	30
28	K-4	II	2003	Moscow,	CC 5	25	30	29	24	40	30	37	34	37	15	38	28	21	25	26	28	34	26	30	44	36	40	36	22
29	GIMC2036:LmcM56	II	2019	Moscow,	8	22	21	19	22	20	29	29	18	15	19	27	22	27	21	35	24	24	17	18	31	34	26	22	36
27	M65	II	2019	Moscow,	321	22	24	20	23	44	32	28	20	18	21	27	18	27	23	20	30	28	20	21	32	28	26	22	
31	GIMC2042:LmcM63	II	2019	Moscow,	121	22	20	16	25	30	29	27	20	21	21	29	23	29	23	28	27	28	25	22	30	36	29	27	18
32	GIMC2048:LmcC5	II	2020	Moscow,	8	20	24	18	22	30	30	26	18	19	17	24	21	25	22	23	27	26	21	21	32	48	26	21	30
33	GIMC2051:LmcC15	II	2020	Moscow,	1005	23	23	20	*25*	28	29	28	17	18	15	31	23	23	23	28	25	27	24	21	29	32	27	23	25
	GIMC2026:LmcBD14	II	2019	Moscow,	37	22	23	18	*24*	42	27	27	19	14	22	27	20	26	24	22	23	27	19	21	31	44	28	23	24
35	49	II	2019	Moscow,	CC 9	24	28	16	22	32	31	30	27	26	16	31	21	32	23	26	27	34	21	24	35	42	30	27	22

36	48	II	2019	Belarus	CC 8	24	27	18	23	38	30	29	24	23	12	32	21	31	24	21	26	32	21	23	36	36	30	27	22
37	79	II	2019	Tver region	CC 37	20	27	20	24	36	29	30	26	23	17	35	21	30	23	24	26	32	21	23	33	28	32	25	21
38	69	II	2019	Moscow	CC 37	23	26	16	21	30	33	34	27	26	12	34	21	32	25	23	27	34	22	23	36	34	34	27	20
39	84	II	2019	Moscow	CC 37	23	27	18	24	26	29	29	21	21	11	33	19	31	23	22	24	33	21	22	34	36	28	25	17
40	25	II	2019	Bryansk	CC 1	23	24	20	23	30	28	30	23	20	13	31	22	30	22	23	24	31	20	22	33	48	30	26	23
41	70	II	2019	Moscow	CC 8	24	26	18	23	44	30	31	26	23	10	32	18	31	23	20	26	32	20	23	35	32	30	26	20
42	50	II	2019	Moscow	CC 1	22	26	16	24	27	30	31	25	22	13	34	23	33	24	24	27	33	21	23	35	44	32	28	20
43	71	II	2019	Moscow	CC 59	23	26	18	24	40	30	27	23	20	25	28	21	26	22	21	23	25	21	24	36	40	31	27	20
44	80	II	2019	Moscow	CC 37	23	27	18	22	27	28	30	22	20	15	32	25	30	21	22	22	24	21	23	36	33	28	26	24
45	78	II	2019	Tver region	CC 37	23	25	17	22	32	32	29	20	20	14	31	20	31	22	23	23	24	18	22	34	42	30	26	26
46	75	II	2019	Belarus	CC 9	23	25	20	23	28	36	30	24	20	12	32	21	30	23	23	25	32	22	23	35	36	33	26	20
47	30	II	2019	Tambov	CC 18	20	21	24	24	17	28	29	22	24	14	33	23	31	23	23	23	27	20	23	35	34	32	28	15
48	29	II	2019	Tambov	novel	22	24	19	21	21	28	27	22	23	13	30	17	29	24	24	25	33	21	23	35	28	32	27	17
49	42	II	2019	Tambov	CC 18	21	27	20	24	22	29	27	23	23	13	33	19	32	24	19	24	31	22	24	37	36	34	29	25
50	82	II	2019	Moscow	CC 37	22	23	19	23	32	29	28	21	24	14	26	17	25	24	20	27	33	19	22	31	48	30	25	23
51	27	II	2019	Moscow	CC 18	22	26	19	23	29	29	29	21	22	13	33	20	31	23	23	24	31	20	22	35	32	30	24	22
52	GIMC2004:Lmc76_19	II	2019	Moscow	288	22	24	20	24	32	30	30	23	24	23	30	20	30	24	21	32	30	23	20	32	44	29	27	20
53	GIMC2035:Lmc7218	II	2019	Moscow	9	24	30	16	24	22	33	34	25	22	20	26	21	30	25	22	30	30	24	22	34	42	32	19	22
54	GIMC2054:Lmc6888	II	2020	Moscow	37	23	23	18	22	29	26	26	27	15	20	23	21	25	22	23	22	25	19	21	30	36	26	20	16

55	GIMC2053:Lmc6646	II	2020	Moscow	37	28	25	20	23	30	30	30	24	34	26	30	21	30	29	23	30	30	22	20	24	28	30	24	20
56	GIMC2062:Lmc5157	II	2021	Moscow	26	24	24	18	25	24	29	30	22	22	22	27	19	30	25	23	25	27	20	22	30	34	30	23	16
57	GIMC2024:Lmc2689	II	2019	Moscow	155	27	29	16	22	29	33	32	28	25	21	31	22	33	28	24	33	23	28	24	30	36	35	27	28
58	GIMC2044:LmcM65	II	2020	Moscow	121	22	22	18	24	30	33	28	24	16	24	32	18	30	24	24	31	32	26	20	35	48	30	21	28
ANIMAL																	23			20	27					32			
1	2598	II	1960	Kabardino-Balkaria ASSR	CC 7	18	18	19	23	39	29	27	23	21	21	32	21	30	24	23	26	25	21	22	29	44	32	18	27
2	39 ГНКИ	II	1956	Irkutsk region	CC 7	23	17	23	24	36	40	32	29	24	19	35	25	34	26	22	27	30	22	25	38	40	34	30	22
3	K-17	II	1956	Ukrainian SSR	CC 19	28	30	30	24	28	35	30	39	13	24	33	20	32	24	24	23	34	21	23	34	33	34	25	28
4	97	II	1960	Voronezh region	CC 124	24	24	26	22	29	36	32	32	20	24	33	21	33	23	24	26	26	22	23	29	42	33	24	24
5	27-T	II	1966	Tajik ASSR	CC 7	16	16	24	23	30	37	29	23	0	19	31	23	32	26	19	24	25	20	24	28	36	37	22	24
6	134	II	1952	Moscow region	CC 7	20	21	22	22	34	34	28	30	33	18	34	17	32	26	21	26	23	21	24	29	33	33	22	25
7	944	II	1958	Moscow region	CC 7	20	18	21	23	26	40	31	33	30	19	35	19	33	25	19	26	25	22	25	37	35	32	21	22
8	4-40	II	1947	Moscow	CC 7	20	24	23	24	30	39	30	30	31	19	31	22	30	23	17	25	32	19	22	30	37	31	20	23
9	3501	II	1965	Moscow	CC 7	18	18	22	21	30	40	30	28	30	12	31	22	32	22	17	25	25	20	23	30	30	35	17	24
10	3880	II	1970	Tyumen region	CC 7	21	17	25	24	32	36	31	30	30	14	34	22	35	24	22	25	10	23	25	36	33	32	23	23
11	1-CAX	II	1971	Sakhalin region	CC 7	17	16	23	23	28	39	32	28	34	13	34	17	33	23	19	24	28	24	23	37	33	37	20	26
12	1-67	II	1972	Altai region	CC 7	16	15	18	23	29	40	30	30	25	24	35	12	33	30	22	26	25	21	24	38	30	38	20	22
13	2-П	II	1967	Bashkir ASSR	CC 177	18	15	22	24	26	36	30	25	32	19	31	20	31	24	23	26	28	20	24	38	33	32	20	21
14	3-П	II	1966	Bashkir ASSR	CC 7	20	17	22	24	28	39	30	30	28	18	34	21	33	24	20	25	30	20	23	39	31	33	24	27
15	4-Г	II	1970	Kazakh ASSR	CC 7	18	14	20	22	30	40	30	23	30	14	33	19	32	22	19	25	27	20	23	39	32	32	26	25

16	33	II	1975	Chita region	CC 7	17	18	18	23	32	38	28	32	30	12	34	17	32	33	21	25	27	21	24	37	31	32	20	31
17	45	II	1971	Kazakh ASSR	CC 307	16	17	21	25	32	39	30	30	30	19	34	15	33	26	20	27	25	22	24	35	31	33	15	25
18	7-B	II	1972	Brest region (Byelorussian SSR)	CC 18	20	20	22	22	32	37	30	30	30	24	32	20	34	27	20	25	25	23	25	36	33	32	17	20
19	211	II	1992	Kursk region	CC 7	25	22	26	22	27	34	31	32	30	20	31	23	34	27	25	22	32	21	23	37	34	37	25	25
20	Chisto-pol	II	1964	Tatar ASSR	CC 7	18	26	22	21	32	40	32	33	34	19	29	20	32	22	18	25	23	23	24	38	31	38	23	27
21	119	II	1967	Udmurt ASSR	CC 177	23	22	25	23	29	39	30	30	33	18	32	23	33	24	33	26	30	20	24	38	34	32	22	27
22	178-P	II	1967	Uzbek SSR	CC 7	20	17	20	21	32	40	29	25	0	18	33	24	33	24	15	23	24	21	23	39	31	33	20	25
23	170	II	1974	Khabarovsk re-gion	CC 177	25	22	25	22	22	36	30	33	34	15	33	23	31	25	21	27	30	20	24	39	32	32	22	27
24	816-D	II	1975	Dagestan ASSR	CC 21	20	16	25	22	29	37	29	34	33	23	31	21	35	24	20	24	28	20	25	37	33	32	16	25
25	1426	II	1970	Irkutsk region	CC 101	20	18	22	23	30	37	31	36	26	20	34	16	33	23	18	26	26	21	25	35	31	33	20	26
26	257	II	1971	Novgorod re-gion	CC 124	20	22	25	24	24	36	33	40	26	20	34	22	33	30	22	26	35	22	23	36	32	33	16	26
27	406	II	1964	Kazan	CC 7	26	28	32	21	29	36	30	36	36	26	35	30	31	24	22	25	30	21	23	37	31	32	28	28
28	197	II	1955	Ukrainian SSR	CC 7	22	20	22	24	30	37	30	38	38	21	31	20	33	24	22	25	31	21	22	38	31	32	28	27
29	15	II	1952	Belorussian SSR	CC 7	28	24	25	23	26	34	28	33	32	12	34	21	32	22	14	25	28	21	23	38	32	31	27	26
30	14-P	II	1969	Altai region	CC 7	26	23	26	23	39	36	29	33	34	14	33	25	32	22	22	27	27	20	23	39	32	32	22	26
31	6	II	1952	Ryazan Oblast	CC 18	28	26	30	24	36	33	27	36	32	19	34	26	30	26	27	25	35	21	25	39	33	30	28	26
32	3453	II	1965	Moscow	CC 7	20	22	25	24	28	35	30	30	33	18	34	28	31	22	20	22	28	20	21	37	32	33	24	28
33	191-08	II	2008	Tver region	CC 18	22	16	27	22	29	33	27	36	25	15	34	24	33	24	25	25	26	21	25	35	30	33	25	27
34	45	II	1957	Ukrainian SSR	CC 7	26	22	25	23	22	35	30	32	41	12	34	28	31	23	24	24	34	20	24	36	34	32	23	29
35	174	II	1971	Brest region (Byelorussian SSR)	CC 124	26	28	30	25	32	40	29	35	34	12	34	24	32	25	27	25	36	21	25	37	33	34	24	25

36	«A»	II	1952	Kazakh ASSR	CC 124	22	22	29	22	29	37	27	33	33	17	33	24	31	25	24	24	30	21	23	36	32	33	23	24
37	121	II	1964	Moscow region	CC 124	24	18	28	21	32	34	31	32	35	12	34	24	33	24	24	25	33	22	23	38	33	34	26	23
38	35	II	1962	Kazakh ASSR	CC 101	20	25	22	22	22	36	26	30	27	19	35	17	33	26	20	25	21	21	23	35	32	30	21	25
39	382	II	1954	Yaroslav region	CC 18	23	21	28	21	29	33	27	32	25	17	31	25	30	22	24	22	30	22	21	37	33	29	27	24
40	324	II	1965	Moscow	CC 7	22	19	24	23	30	35	26	31	19	28	34	23	31	24	26	25	30	21	23	35	31	29	25	24
41	50	II	1971	Brest region (Byelorussian SSR)	CC 7	21	21	20	22	24	33	28	35	0	18	33	20	33	23	23	24	30	21	21	38	32	30	20	24
HUMAN												26											21	22					
1	57	II	1971	Moscow	CC 7	30	25	32	21	30	40	28	36	34	18	33	31	29	23	26	23	34	20	22	33	31	30	22	25
2	29 ч	II	1999	Tula region	CC 177	25	20	24	22	27	36	28	31	33	20	32	23	34	24	24	24	30	21	24	36	32	31	25	27
3	5ч	II	1975	Tula region	CC 7	30	28	30	21	39	39	25	42	30	17	30	32	31	20	34	22	38	20	22	32	32	29	31	24
4	24618	II	1971	Moscow	CC 7	24	20	25	24	36	33	27	32	33	19	27	24	33	21	24	22	33	20	22	33	34	29	24	24
5	K-23	I	1988	Moscow	CC 1	17	20	21	21	28	34	29	28	36	18	32	17	31	24	23	25	25	20	24	35	32	32	22	25
6	GIMC2009:LmcUH4	II	2019	Moscow	7	15	22	20	24	29	28	26	20	17	16	27	23	24	20	23	23	27	19	23	30	33	26	23	23
7	GIMC2010:LmcUH8	II	2019	Moscow	7	23	23	16	23	30	29	27	16	16	12	30	20	30	22	20	23	29	20	21	35	32	30	25	24
8	GIMC2013:LmcUH16	I	2019	Moscow	6	18	23	18	23	34	30	26	18	15	14	27	23	29	22	19	21	29	18	22	26	32	29	24	26
9	GIMC2029:LmcUH18	II	2019	Moscow	155	28	28	20	24	26	31	28	19	19	12	28	24	30	22	21	21	30	20	23	33	33	30	24	25
10	GIMC2030:LmcUH19	I	2019	Moscow	6	20	23	18	24	30	29	25	17	19	13	27	23	25	21	20	21	30	20	21	28	31	29	24	27
11	GIMC2028:LmcUH17	II	2019	Moscow	7	24	23	19	22	24	26	25	19	15	20	29	21	28	20	20	24	25	19	21	33	30	27	25	19

12	GIMC2031:LmcUH20	II	2019	Moscow	2096	22	23	18	23	20	28	27	20	17	17	27	16	27	22	25	24	25	18	22	32	33	25	22	20
13	GIMC2032:LmcINH-1	II	2019	Moscow	7	38	24	20	25	23	30	32	25	18	28	30	22	30	24	18	30	30	26	22	38	34	30	27	24
14	GIMC2060:LmcH24-1	II	2021	Moscow	451	20	30	18	22	26	28	28	21	18	20	28	30	27	23	33	23	28	19	22	31	31	27	24	23
15	GIMC2056:LmcEH-1	II	2020	Moscow	21	23	23	28	25	21	28	25	19	16	24	28	20	30	22	25	24	28	21	22	30	34	30	24	27
16	GIMC2055:LmcIH1-4	I	2020	Moscow	4	24	25	23	22	25	32	30	28	35	23	30	21	30	23	21	26	30	24	22	24	31	26	25	21
17	H67 -1	I	2019	Moscow	6	19	30	23	21	21	24	23	20	15	20	28	25	27	25	20	27	28	22	22	25	32	27	23	23
18	GIMC2034:LmcH67-3	I	2019	Moscow	5	23	21	22	22	22	26	23	17	16	14	29	22	29	26	18	25	26	18	20	23	33	25	24	22
19	GIMC2058:LmcH67-4	I	2020	Moscow	1	24	18	19	21	20	30	28	24	18	24	30	24	30	24	22	30	30	24	24	30	32	23	26	21
20	GIMC2014:LmcSH3	II	2018	Moscow	14	22	24	21	*24*	21	30	30	18	20	24	27	22	30	24	22	24	28	23	20	32	32	30	25	22
